# International links between *Streptococcus pneumoniae* vaccine serotype 4 sequence type (ST) 801 in Northern European shipyard outbreaks of invasive pneumococcal disease

**DOI:** 10.1016/j.vaccine.2021.10.046

**Published:** 2022-02-11

**Authors:** R.A. Gladstone, L. Siira, O.B. Brynildsrud, D.F. Vestrheim, P. Turner, S.C. Clarke, S. Srifuengfung, R. Ford, D. Lehmann, E. Egorova, E. Voropaeva, G. Haraldsson, K.G. Kristinsson, L. McGee, R.F. Breiman, S.D. Bentley, C.L. Sheppard, N.K. Fry, J. Corander, M Toropainen, A. Steens, The Global Pneumococcal Sequencing Consortium, Patrick E Akpaka, Krow Ampofo, Martin Antonio, Veeraraghavan Balaji, Bernard W. Beall, Houria Belabbès, Rachel Benisty, Godfrey Bigogo, Abdullah W Brooks, Philip E. Carter, Jennifer E. Cornick, Alejandra Corso, Maria Cristina de Cunto Brandileone, Samanta Cristine Grassi Almeida, Nicholas J. Croucher, Ron Dagan, Alexander Davydov, Idrissa Diawara, Sanjay Doiphode, Mignon du Plessis, Naima Elmdaghri, Özgen Köseoglu Eser, Dean B. Everett, Diego Faccone, Paula Gagetti, Noga Givon-Lavi, Md Hasanuzzaman, Paulina A. Hawkins, Waleria Hryniewicz, Kristina G. Hulten, Margaret Ip, Aurelie Kapusta, Rama Kandasamy, Tamara Kastrin, Jeremy Keenan, Keith P. Klugman, Brenda Kwambana-Adams, Pierra Y. Law, John A Lees, Pak Leung Ho, Yuan Li, Stephanie W. Lo, Theresa J. Ochoa, Shabir A. Madhi, Benjamin J Metcalf, Jennifer Moïsi, Helio Mucavele Fundação Manhiça, Kedibone M. Ndlangisa, Michele Nurse-Lucas, Susan A. Nzenze, Stephen K Obaro, Metka Paragi, Andrew J Pollard, KL. Ravikumar, Ewa Sadowy, Samir K. Saha, Eric Sampane-Donkor, Shamala Devi Sekaran, Sadia Shakoor, Shrijana Shrestha, Betuel Sigauque, Anna Skoczynska, Kwan Soo ko, Peggy-Estelle Tientcheu, Leonid Titov, Yulia Urban, Jennifer Verani, Andries J. van Tonder, Anne von Gottberg, Nicole Wolter

**Affiliations:** v Department of Paraclinical Sciences, The University of the West Indies, St. Augustine, Trinidad and Tobago; w Division of Pediatric Infectious Diseases, Department of Pediatrics, School of Medicine, University of Utah, 295 Chipeta Way, PO BOX 581289, Salt Lake City, UT, 84108, USA; x WHO Collaborating Centre for New Vaccines Surveillance, Medical Research Council Unit The Gambia at The London School of Hygiene & Tropical Medicine, Fajara, The Gambia; y Christian Medical College, Vellore, India; bv Centers for Disease Control and Prevention, Atlanta, USA; z Ibn Rochd university-hospital center-Casablanca; aa The Faculty of Health Sciences, Ben-Gurion University of the Negev, Beer-Sheva, Israel; ab Centre for Global Health Research, Kenya Medical Research Institute, Kisumu, Kenya; ac International Centre for Diarrheal Diseases Research, Dhaka, Bangladesh; ad Institute of Environmental Science and Research Limited, Kenepuru Science Centre, Porirua, Zealand; ae Malawi-Liverpool-Wellcome-Trust, Malawi; af Instituto Nacional de Enfermedades Infecciosas, Argentina; ag Center of Bacteriology, Adolfo Lutz Institute, São Paulo, Brazil; ah Faculty of Medicine, School of Public Health, Imperial College London, UK; ai Belarusian State Medical University, Minsk, Belarus, The Republican Research and Practical Center for Epidemiology and Microbiology, Minsk, Belarus; aj Faculty of Sciences and Techniques of Health, Mohammed VI University of Health Sciences (UM6SS); ak Hamad Medical Corporation, Doha, Qatar; al Centre for Respiratory Diseases and Meningitis, National Institute for Communicable Diseases, Johannesburg, SouthAfrica; am Laboratoire of Microbiology, Faculty of Medicine and Pharmacy & Ibn Rochd University Hospital Center, Casablanca, Morocco; an Hacettepe University Faculty of Medicine, Department of Medical Microbiology, 06100, Ankara, Turkey; ao Queens Research Institute, University of Edinburgh, UK; ap Instituto Nacional de Enfermedades Infecciosas, Argentina; aq Child Health Research Foundation, Dhaka, Bangladesh; bw Rollins School Public Health, Emory University, USA; ar National Medicines Institute, Division of Clinical Microbiology and Infection Prevention, Warsaw, Poland; as Department of Pediatrics, Baylor College of Medicine, Houston TX; at Dept of Microbiology, Chinese Univ of Hong Kong; au Department of Human Genetics, University of Utah, 15 North 2030 East, Salt Lake City, UT 84112; av Oxford Vaccine Group, Department of Paediatrics, University of Oxford, and the NIHR Oxford Biomedical Research Centre, Oxford, UK; aw Department of Medical Microbiology, Institute of Public Health of the Republic of Slovenia, Grabloviceva 44, 1000 Ljubljana, Slovenia; ax Francis I. Proctor Foundation, University of California, San Francisco, San Francisco, California, United States of America; bx Department of Health Security, Finnish Institute for Health and Welfare (THL), Helsinki, Finland; ay NIHR Global Health Research Unit on Mucosal Pathogens, Division of Infection and Immunity, University College London, London, UK; az Department of Microbiology and Carol Yu Centre for Infection, The University of Hong Kong, Queen Mary Hospital, Hong Kong, China; by Parasites and Microbes, Wellcome Sanger Institute, Cambridge, UK; ba Instituto de Medicina Tropical, Universidad Peruana Cayetano Heredia, Lima, Peru; bb Medical Research Council: Respiratory and Meningeal Pathogens Research Unit, University of the Witwatersrand, SouthAfrica; bc Dept. of Science and Technology/National Research Foundation: Vaccine Preventable Diseases, University of the Witwatersrand, South Africa; bd Agence de Médecine Préventive, Paris, France; be Centro de Investigação em Saúde da Manhiça (CISM), Maputo, Moçambique; bf Department of Paraclinical Sciences, The University of the West Indies, St. Augustine, Trinidad and Tobago; bg University of Nebraska Medical Center, Omaha, USA; bh National Laboratory of Health, Environment and Food, Centre for Medical Microbiology, Department for Public Health Microbiology, Grablovičeva 44, 1000, Ljubljana, Slovenia; bi Central Research Laboratory, Department of Microbiology, Kempegowda Institute of Medical Sciences Hospital & Research Center, Bangalore, India; bj Department of Molecular Microbiology, National Medicines Institute, 00-725 Warsaw, Poland; bk Department of Medical Microbiology, School of Biomedical and Allied Health Sciences University of Ghana, Accra, Ghana; bl Faculty of Medical & Health Sciences, UCSI University, Malaysia; bm Department of Pathology and Laboratory Medicine and Department of Paediatrics and Child Health, The Aga Khan University, Karachi 74800, Pakistan; bn Patan Academy of Health Sciences, Kathmandu, Nepal; bo Fundação Manhiça / Centro de Investigação em Saúde da Manhiça (CISM), Maputo, Mozambique; bp Instituto Nacional de Saúde, Ministério de Saúde, Maputo, Mozambique; bq National Reference Centre for Bacterial Meningitis, Department of Epidemiology and Clinical Microbiology, National Medicines Institute, Warsaw, Poland; br Department of Molecular Cell Biology, Samsung Biomedical Research Institute, Sungkyunkwan University School of Medicine, Suwon, South Korea; bs Vaccines and Immunity Theme, MRC Unit, The Gambia; bt The Republican Research and Practical Center for Epidemiology and Microbiology, Minsk, Belarus; bz G.N. Gabrichevsky Research Institute for Epidemiology and Microbiology, Moscow, Russia; bu Department of Veterinary Medicine, University of Cambridge, Cambridge, UK; a Department of Biostatistics, Institute of Basic Medical Sciences, University of Oslo, Oslo, Norway; b Parasites and Microbes, Wellcome Sanger Institute, Cambridge, UK; c Department of Health Security, Finnish Institute for Health and Welfare (THL), Helsinki, Finland; d Division of Infection Control and Environmental Health, Norwegian Institute of Public Health, Oslo, Norway; e Centre for Tropical Medicine and Global Health, Nuffield Department of Medicine, University of Oxford, Oxford, UK; f Cambodia Oxford Medical Research Unit, Angkor Hospital for Children, Siem Reap, Cambodia; g Faculty of Medicine and Institute of Life Sciences, University of Southampton, UK; h NIHR Southampton Biomedical Research Centre, University Hospital Southampton NHS Trust, Southampton, United Kingdom; i Global Health Research Institute, University of Southampton, Southampton, United Kingdom; j School of Postgraduate Studies, International Medical University, Kuala Lumpur, Malaysia; k Centre for Translational Research, IMU Institute for Research, Development and Innovation (IRDI), Kuala Lumpur, Malaysia; l Faculty of Pharmacy, Siam University, Bangkok, Thailand; m Papua New Guinea Institute of Medical Research, PO Box 60, Goroka 441, Eastern Highlands Province, Papua New Guinea; n Telethon Kids Institute, the University of Western Australia, Perth, WA, Australia; o G.N. Gabrichevsky Research Institute for Epidemiology and Microbiology, Moscow, Russia; p Department of Clinical Microbiology, Landspitali – The National University Hospital of Iceland, Reykjavik, Iceland and Faculty of Medicine, University of Iceland; q Centers for Disease Control and Prevention, Atlanta, USA; r Emory Global Health Institute, Atlanta, USA; s Rollins School Public Health, Emory University, USA; t Vaccine Preventable Bacteria Section, Public Health England - National Infection Service, London, United Kingdom; u Immunisation and Countermeasures Division, Public Health England - National Infection Service, London, United Kingdom

**Keywords:** *Streptococcus pneumoniae*, Pneumococcal, Whole genome sequencing, Outbreak, ST801, Molecular epidemiology, Serotype 4, PCVs, PPV23

## Abstract

**Background:**

Pneumococcal disease outbreaks of vaccine preventable serotype 4 sequence type (ST)801 in shipyards have been reported in several countries. We aimed to use genomics to establish any international links between them.

**Methods:**

Sequence data from ST801-related outbreak isolates from Norway (n = 17), Finland (n = 11) and Northern Ireland (n = 2) were combined with invasive pneumococcal disease surveillance from the respective countries, and ST801-related genomes from an international collection (n = 41 of > 40,000), totalling 106 genomes. Raw data were mapped and recombination excluded before phylogenetic dating.

**Results:**

Outbreak isolates were relatively diverse, with up to 100 SNPs (single nucleotide polymorphisms) and a common ancestor estimated around the year 2000. However, 19 Norwegian and Finnish isolates were nearly indistinguishable (0–2 SNPs) with the common ancestor dated around 2017.

**Conclusion:**

The total diversity of ST801 within the outbreaks could not be explained by recent transmission alone, suggesting that harsh environmental and associated living conditions reported in the shipyards may facilitate invasion of colonising pneumococci. However, near identical strains in the Norwegian and Finnish outbreaks does suggest that transmission between international shipyards also contributed to those outbreaks. This indicates the need for improved preventative measures in this working population including pneumococcal vaccination.

## 1 Introduction

Outbreaks of invasive pneumococcal disease (IPD) in shipyard workers have been observed in multiple European countries in recent years; Northern Ireland (2015), Norway (2019), Finland (2019) and France (2020) [1], [2], [3], [4]. These European outbreaks all involved serotype 4 though other serotypes were also reported (3, 8, 9 N and 12F) [1], [2], [3], [4]. Serotype 4 is included in both the pneumococcal polysaccharide vaccine (PPV23) that is often recommended for older adults and pneumococcal conjugate vaccines (PCVs) routinely administered in the national childhood immunisation programs in most European nations, including the above-mentioned countries. PCV herd effects were quickly reported to have reduced the incidence of IPD of serotype 4 in the adult population [5], [6]. Only four years after PCV7 introduction in England and Wales the incidence rate ratio of serotype 4 was 0.26 for the age group 5–65 years [5]. As such, serotype 4 is not a major cause of IPD in the adult working-age group (Table 1). Multi-locus sequence type (MLST) for IPD surveillance data were available for Northern Ireland, Norway and Finland; sequence types ST801 and ST205 were implicated in serotype 4 IPD in all three locations. For the Northern Irish, Norwegian and Finnish outbreaks, ST801 expressing serotype 4 was common to all and represented the majority of outbreak isolates. This ST was first reported in pubMLST.org in 2001, isolated in the Czech Republic, and was only observed twice (Russia, 2011) in a published international pneumococcal dataset of 13,454 genomes [7].

**Table 1:** 2018 IPD incidence per 100,000 in the working adult population.

Country	Serotype 4	All serotypes	Age group
Finland	0.48	10.3	20–64
Norway	0.23	6.53	20–64
England	0.07	7.18	20–64

The majority of the shipyard workers from the outbreaks were reported to be directly involved in metal welding or worked in interior outfitting, however the cases represented a number of different professions beyond welders. Welders have been documented to be at increased risk of pneumonia and IPD, which may be a consequence of inhalation of metal fumes, further compounded by smoking [8], [9], [10], [11], [12], [13], [14]. Public Health England (PHE) recommends that welders be vaccinated with a single dose of PPV23, whilst the Norwegian Institute of Public Health (NIPH) recommends the individual assessment of the need to vaccinate welders specifically [15]. In Finland, general legislation requires employers to offer vaccinations to protect their employees from occupational infectious diseases hazards. Shipyard workers are a large international community of workers; an outbreak in France in 2020 reported 5,823 people of 102 different nationalities involved in a single shipyard project [4]. Workers are usually housed in densely populated, temporary accommodation arrangements such as barracks and ships, including the renovation projects themselves [4], which can facilitate transmission. In addition, workers move internationally between shipyards where their skills are required which could seed further outbreaks in other shipyards. Combined, these factors warrant an international approach to investigation, management of outbreaks in this community and preventative vaccination of the workforce [16].

Whole genome sequencing offers enhanced resolution beyond ST for determining if isolates are closely related and can help resolve whether the IPD outbreaks were a result of I) recent transmission of a potentially more virulent strain, or II) represent independent causes of IPD from a more genetically diverse group of pneumococci circulating in the population with increased risk for developing severe disease. It is possible to further estimate when isolates may have shared a common ancestor and identify the regions of the world with which a genotype may be associated. As serotype 4 ST801 was confirmed to be involved in at least three separate outbreaks in European shipyards, we sought to examine the genomic relationships between these outbreak strains and, by including additional international isolates of ST801 and related sequence types, to provide a phylogeographical and temporal context for the outbreaks.

## 2 Methods

### 2.1 Outbreak case definitions

The NIPH defined the Norwegian outbreak cases as: individuals with date of symptom-onset from January 2019, having resided in Møre and Romsdal county and either, being confirmed with serotype 4 IPD (confirmed cases), or working at the specific shipyard AND having clinical symptoms compatible with lower respiratory tract infection or IPD but without microbiological confirmation, OR having serotype 4 *Streptococcus pneumoniae* isolated from non-sterile material (e.g. nasopharynx swab) (probable cases). Six confirmed and ten probable cases were directly linked to the primary shipyard, the connection was uncertain for the remaining four confirmed cases, of which two could be generically linked to the shipyard industry in the area [2]. No other cases/serotypes were observed in IPD surveillance in the area.

The Public Health Agency, Health Protection Service, Northern Ireland (PHA-NI) defined outbreak cases as: individuals who worked at the Belfast shipyard after 11th January 2015 AND for a confirmed case: a clinical diagnosis of IPD or pneumococcal pneumonia AND at least one of the following: *S. pneumoniae* isolated from a normally sterile site, pneumococcal DNA or antigen detected in fluid from a normally sterile site or pneumococcal antigen detected in urine. For a probable case: a clinical presentation compatible with IPD (conditions such as meningitis or empyema) or pneumonia (supported by radiographic imaging) where serious pneumococcal disease based on available clinical, microbiological and epidemiological evidence is the most likely diagnosis, in the absence of laboratory confirmation [3]. The outbreak was declared over in July 2015. Four confirmed cases and five probable cases were identified; of the confirmed cases, two were determined to be serotype 4 ST801, one serotype 4 ST205 and one serotype 3 isolate [3].

The Finnish Institute for Health and Welfare (THL) defined outbreak cases as individuals who had worked at the shipyard after 1st February 2019 and presented with a clinical diagnosis consistent with IPD or pneumococcal pneumonia and (for a confirmed case) had *S. pneumoniae* isolated from blood or cerebrospinal fluid or pneumococcal antigen detected in urine. If there was no laboratory confirmation, the case was defined as probable [1]. The outbreak was declared over in November 2019. Altogether 31 confirmed cases and six probable cases were identified. Twenty-five cases were serotyped of which 11 were serotype 4, 13 serotype 12F and one serotype 8 [1].

### 2.2 Data selection

This analysis was restricted to isolates related to ST801 which was common to the three IPD outbreaks in shipyard workers in Norway, Finland and Northern Ireland. It was previously reported that ST801 was a member of the Global Pneumococcal Sequencing Cluster (GPSC)162 [7]. We therefore screened over 40,000 international pneumococcal genomes for isolates belonging to GPSC162. These international genomes had been sequenced on Illumina HiSeq and X10 platforms at the Wellcome Sanger Institute and had been assigned to a GPSC using PopPUNK and represented multiple carriage and/or disease collections sampled in Europe, Africa, Asia, Oceania and the Americas [17]. A list of STs that the relevant GPSC162 isolates represented was collated. Subsequently, the outbreak countries screened their available IPD genomes for all known STs within GPSC162 in addition to single locus variants of ST801 listed in pubMLST (accessed February 2020), n = 21. IPD isolates from surveillance are routinely sequenced at all three public health institutes, although the Norwegian data for 2018–2019 are incomplete.

### 2.3 Bioinformatics

Assembly and annotation was performed using Shovill and Prokka as part of the Nullabor package [18], [19]. GPSC and ST were assigned and serotype was inferred using Pathogenwatch [20]. As incomplete antimicrobial susceptibility testing data were available for the combined datasets and to provide a single standardised method, antimicrobial resistance was inferred using Pathogenwatch [20].

A published Global Pneumococcal Sequencing project (GPS) assembly of ST801 was selected as a draft reference (GCA_90129732) [21]. Contigs of < 500 bp were removed (7/50) and reordered against a completed reference genome (ATCC700669) using ABACAS v1.3.1 and concatenated into a final length of 2,051,095 bp [22]. The included GPSC162 genomes were mapped against the reference using Snippy v4.6.0 and the resultant alignment input into Gubbins v2.4.0 to identify recombination and produce a RAxML v8.2.8 recombination free phylogeny [19], [23], [24]. PairSNP v0.2.0 was used to calculate pairwise SNPs distances between the included genomes from the SNP sites Gubbins alignment [25]. The pangenome was defined using Panaroo v1.0.2 [26]. Data were visualized in Microreact and Phandango [27], [28]. Fastq data used in this study are deposited in the European Nucleotide Archive (ENA) and accessions are included in the supplement.

A phylogenetic temporal analysis was performed to estimate the dates of common ancestors within the GPSC162 tree. Gubbins output was supplied to the BactDating R package v1.0 in three replicates and one with randomised tip dates. These ran through the Markov Chains Monte Carlo algorithm using 100,000,000 generations sampled every 100,000 states with a 10,000,000 burn-in using the mixed gamma model [29]. The three replicate MCMC chains were deemed to have converged with Gelman diagnostic of approximately 1 for mu, sigma and alpha using the coda R package [30]. We assessed whether the effective sample size (ESS) on the first replicate model was>200 using the effectiveSize function of the coda R package [30]. The randomised dates model did not converge.

The presence and absence of virulence genes was determined as part of the Nullabor package using the virulence factor database (VFDB) [19], [31]. PANINI was used to visualise clustering of the accessory gene content [32]. Scoary with no pairwise comparisons was used to determine which genes were associated with CC801 compared to CC4127 [33].

### 2.4 Consent statement

All information regarding the isolates used in this study was anonymised before analysis. Appropriate approvals for the use of isolates were obtained from each institution contributing unpublished genomes. No tissue material or other biological material was obtained from humans.

## 3 Results

### 3.1 Available data

Illumina sequence data was available from NIPH for the 17 Norwegian outbreak isolates belonging to clonal complex (CC)801 isolated in January-April 2019 [2] and a further five CC801 isolates from routine IPD surveillance between 2005 and 2018. Illumina sequence data were available from PHE for the two ST801 Northern Ireland outbreak strains identified between April-June 2015 [3] and a further 16 CC801 from surveillance in England between 2015 and 2019. Illumina sequence data were obtained from THL for the 11 CC801 Finnish outbreak isolates identified between May-November 2019 [1] and a further 14 CC801 isolates from surveillance between 2018 and 2019.

Screening of the international dataset of over 40,000 pneumococcal genomes for GPSC162 identified 41 relevant genomes: 14 had previously been published as part of the GPS project [7], a further 24 had since been sequenced as part of ongoing GPS work (unpublished), two isolates were identified in the Mae La carriage study [34] and one was identified in an IPD study in Iceland (unpublished). Together, a total of 106 genomes were available for the analysis; their complete metadata are included in the supplementary tables.

### 3.2 Geographical and temporal distribution of GPSC162

The 106 genomes were isolated in 12 different countries representing Europe, Asia, Oceania and Africa. The genomes fell into two major clades representing CC4127 (n = 33) and CC801 (n = 73) all of which were inferred to be serotype 4. CC4127 isolates were almost all (32/33) isolated in South East Asia and Oceania and included a few carriage isolates (6/33) while almost all CC801 isolates were isolated in countries in and around northern Europe and only from IPD. These two major clades were estimated to have diverged around the year 1759 [1560–1885] by our phylogenetic dating. An interactive view of the GPSC162 phylogeny with overlaid metadata is available in Microreact https://microreact.org/project/gpsGPSC162.

**Fig. 1:**
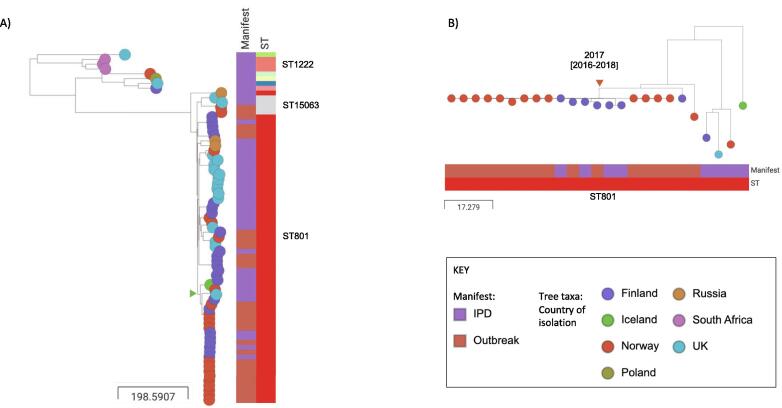
Phylogeny of CC801 and outbreak clade, Fig. 1A) Phylogeny of CC801 n = 73, SNP scale. Green triangle denotes subtree that is expanded in Fig. 1B) Subtree of closely related isolates n = 25, SNP scale. Most recent common ancestor (MRCA) of the cluster containing n = 19 near indistinguishable Norwegian and Finnish isolates (Dark orange triangle). Available in Microreact views https://microreact.org/project/gpsGPSC162/dce8ac7c and https://microreact.org/project/gpsGPSC162/fdf71de9.

Despite screening available IPD surveillance genomes in Norway, Finland and Northern Ireland for all known STs within GPSC162, all belonged to the CC801 clade. ST801 isolates from Iceland (n = 1) Poland (n = 1) and Russia (n = 3) were also identified from the international collection. Only three related isolates of ST1222 from South Africa, found near the root of the CC801 clade, represented a different geographical region for CC801 (Fig. 1A). The whole of CC801 was predicted from the genomic data to be pan-susceptible to clinically relevant classes of antibiotics, this matched the phenotypic profiles that were available (full details in Supplementary metadata and visualised in Microreact https://microreact.org/project/gpsGPSC162/9ba9b178). For the 56/106 samples for which penicillin susceptibility data was available, all were sensitive. A single known pneumococcal resistance determinant was detected in one isolate from Russia, the *tet* gene conferring tetracycline resistance. Overall, within CC801, the ST801 isolates (n = 61/73) represented a diverse group with a max SNP distance of 123. The outbreak isolates were almost exclusively ST801 with 2/30 being the single locus variant ST15063 observed in the Norwegian outbreak. The maximum pairwise SNP distance for the 30 outbreak isolates from the three countries was 100 (Supplementary). In the phylogenetic temporal analysis we estimated that the ST801 isolates within CC801 had shared a common ancestor in 1994 [1980–2002], whilst the common ancestor of all the outbreak isolates was in 2000 [1992–2005].

### 3.3 Norwegian isolates

The maximum SNP distance for the Norwegian outbreak isolates was 96. Of the 17 Norwegian outbreak strains, 13 isolates were virtually indistinguishable, all ST801 with pairwise SNP distances of 0–1 and no recombination detected. Two of the remaining 4 outbreak isolates were also ST801 but phylogenetically distinct with minimum pairwise SNP distances from the main cluster of Norwegian outbreak isolates of 10 and 70 SNPs. The final two Norwegian outbreak isolates were ST15063, with no detected recombination between them and a pairwise SNP distance of 1 from each other with a minimum. The minimum SNP distance between these ST15063 isolates and any other Norwegian outbreak isolate was 69 and there was additional evidence of recombination. Two isolates of ST15063 were observed in PHE IPD surveillance but differed by at least 17 SNPs from the Norwegian ST15063 isolates. Note that the two ST15063 isolates and the two ST801 isolates that did not belong to the cluster of 13 were isolated during the first two months of the outbreak. The five Norwegian isolates from surveillance (ST801 = 4, ST3758 = 1 - the latter from 2007) did not immediately cluster with any Norwegian outbreak isolate; the minimum SNP distance between a surveillance and outbreak strain was 43 SNPs.

### 3.4 Northern Ireland outbreak strains

The two ST801 2015 outbreak isolates from Northern Ireland were indistinguishable by SNPs or recombination. The closest isolate to these outbreak strains was a single Norwegian outbreak isolate from 2019 with a pairwise SNP distance of 7 whereas the closest PHE isolate from routine WGS surveillance in England was isolated in 2018 and had a 48 pairwise SNP distance.

### 3.5 Finnish outbreak isolates

The maximum SNP distance for the 11 Finnish outbreak isolates was 89, these were found in five distinct groupings when using a pairwise SNP threshold of 15. In three of these five groupings Finnish surveillance isolates were also found, and three Finnish surveillance isolates (one singleton and one pair) had a SNP distance of only one to a Finnish outbreak isolate.

### 3.6 Shared outbreak cluster

Six Finnish isolates (two from the outbreak and four from surveillance) were closely related to the cluster of 13 Norwegian outbreak isolates (0–2 SNPs). Outbreak isolates from Northern Ireland were not part of this cluster. Three of the four from Finnish surveillance were diagnosed in the same hospital district as the shipyard and in the same time period as the outbreak (summer-autumn 2018) though no link to the shipyard was identified. There was also no evidence of recombination differences between these isolates and limited differences in accessory gene content, where hypothetical genes and transposases accounted for the vast majority of missing genes in the Finnish isolates compared to the Norwegian isolates. The 19 closely related isolates from Norway and Finland were estimated to share a common ancestor in 2017 [2016–2018] from the phylogenetic temporal analysis (Fig. 1B).

### 3.7 Pangenome analysis

The core genome of GPSC162 consisted of 1661 genes (present in >=99%). The gene content of GPSC162 fell in to two major clusters representing CC4217 and CC801. Of the 875 accessory genes 99 were significantly associated with CC801, 66 of which were hypothetic proteins. A further 231 genes were negatively associated with CC801. Thirty-three different virulence factors were detected in the collection with 20–31 per isolate (mode 24). The virulence gene presence absence tables is included in the supplementary and also presented interactively in Microreact https://microreact.org/project/gpsGPSC162/e3d869c2 along with the accessory gene clustering. The allele percentage identity of ply to the VFDB differentiated CC4127 (99.58%) and CC801 (100%). All of CC4127 and one isolate, basal in the CC801 phylogeny, shared a lytC allele with 100% identity to the allele in the VFDB. The remaining CC801 isolates had a lytC allele with 99.93% identity with the allele in the VFDB. Seventeen of the thirty outbreak isolates analysed had a reduced percentage identity of 83.33% to the pce allele in the VFDB. This allele was shared by 22 of 25 closely related isolates in a subcluster that contained the shared outbreak cluster.

## 4 Discussion

The lineage in which the outbreak strain ST801 was found, GPSC162, was rare in a large international dataset of > 40,000 pneumococcal genomes, collated from various carriage and disease collections sampled across the globe, with only 41 isolates identified. Most of these international GPSC162 isolates were from South East Asia and Oceania and belonged to the CC4127 clade rather than the “Northern European” CC801 in which only 8 of the 41 international isolates fell. The international collection does not represent a balanced sampling of different geographical areas. In spite of its sampling biases it does have considerable sampling (>1000 IPD isolates) from all continents sufficient to declare that CC801 is a rare clone globally, and determine that the few isolates were almost exclusively found in and around the European region. In a recent report on the STs of serotype 4 IPD in adults in the United States, ST801 was not observed, suggesting it does not circulate generally in developed nations [35].

The ST801 isolates represented a diverse group of isolates with a max SNP distance of 123 and the common ancestor was estimated to exist around 1994. The isolates associated with the Norwegian and Finnish outbreak had maximum SNP distances of 96 and 89, respectively, implying that the diversity in these outbreaks and total ST801 diversity is largely overlapping. This level of genetic diversity cannot be explained solely by recent transmission during the outbreak time periods, as they were estimated to share a common ancestor around the year 2000. However, the close phylogenetic relationship of 13/17 Norwegian outbreak isolates with 0–1 SNPs, and a separate pair of identical isolates in the outbreak in Northern Ireland were consistent with a point source outbreak resulting from transmission of a particular strain of ST801 amongst workers. The mutation rate for pneumococci has previously been reported in the region of 1–1.5 SNPs per genome (2 megabase) per year [36]. The overlap between outbreak isolates from Norway and two Finnish outbreak isolates may be explained by international transmission between shipyards via the internationally mobile workforce. The observation of a further 4 closely related IPD cases in Finland could suggest either that these were outbreak cases that were not initially identified as linked to the outbreak, as there was limited ability to identify friends and family members, or transmission in the wider community.

These outbreaks are vaccine preventable however occupational vaccination policies vary between countries, as may implementation and uptake; the majority of the workers in the respective outbreaks were from countries other than Norway (80%, 16/20) or Finland (59%, 22/37), making it difficult to determine if the entire workforce is adequately vaccinated, again indicating a need for an international effort in the prevention of these outbreaks.

We present evidence firstly of transmission of closely related ST801 isolates within an outbreak and potentially between international shipyards, and secondly multiple genetically distinct instances of ST801 causing disease in the outbreaks. The later scenario suggests working conditions are such that there are multiple opportunities for independent IPD cases to arise, supported by the observation of serotype 4 ST205 and other serotypes in the initial reports [1], [3], and that ST801 must be circulating in the workforce or wider community. Those independent colonisation events and direct disease transmission events can be facilitated by crowded living conditions.

The shipyard working population has been shown previously to be at increased risk for severe pneumococcal disease [8], [9], [10], [11], [12], [13], [14]. In addition, serotype 4 is known to be invasive and involved in outbreaks; it was reported to account for 10% (3/29) of outbreaks published between 2000 and 2017 in a systematic review of pneumococcal outbreaks [37]. Furthermore, serotype 4 IPD in adults has previously been reported to be positively associated with smoking [38] and in the Finnish outbreak, the majority of cases were smokers, mostly without underlying conditions [1]. Underlying medical conditions were not common in the Norwegian outbreak, several were smokers and as such smoking was no longer permitted in the shipyard. Serotype 4 has been implicated in recent reports on people experiencing homelessness in the USA with parallels to our finding evidence of both transmission and considerable diversity in clusters of cases [35], [39]. This highlights the propensity of serotype 4 to be associated with outbreaks in at risk adults regardless of clone type, their data suggests an adult reservoir for serotype 4 though the US dataset and the dataset used here suggest carriage is rarely detected. This may be due to short carriage duration which in turn could explain low antimicrobial resistance levels which are typical of serotype 4. Insufficient data exists to determine if ST801 is more invasive than other clones expressing serotype 4 as has been shown for other ST-serotype combinations [7]. A recent study on another European pneumococcal shipyard outbreak also concluded that progression from carriage to invasive disease was facilitated by the conditions associated with shipyards [40].

This study also emphasises the value of the large open database of international genome sequences for determining genomic relationships between the strains and the phylogeographical and temporal context for outbreaks. We initially hypothesised that a strain of ST801 shared between multiple shipyard outbreaks could represent adaptation to this niche. Whilst outbreak strain diversity was similar to ST801 overall it does not rule out that ST801 has an advantage when airways are exposed to harsh conditions. Though we stress that serotype 4 maybe the more defining feature of outbreaks in at risk adults we determined the virulence factors and defining gene content associated with CC801, and highlighted differences in gene context to CC4127. Genomic collections can provide an opportunity to identify whether there have been any specific adaptations which could play a role in the outbreaks. However, further data including sampling of carriage during outbreaks and of the general at risk population (shipyard workers, adults), which is sparse, would be informative to allow for robust sampling and well-designed analyses to capture any causative genetic variation.

The results of this study stress the need for better implementation of preventive measures more broadly in this susceptible working population, as cases were not limited to welders, including pneumococcal vaccination, more stringent and possibly wider use of personal protective equipment during work in confined areas where welding takes place, improved living conditions, promotion of hygiene measures, and stressing the compounding dangers of smoking.

## CRediT authorship contribution statement

**R.A. Gladstone:** Methodology, Data curation, Investigation, Formal analysis, Visualization, Writing – original draft. **L. Siira:** Data curation, Resources, Writing – review & editing. **O.B. Brynildsrud:** Investigation, Data curation, Writing – review & editing. **D.F. Vestrheim:** Writing – review & editing. **P. Turner:** Resources, Writing – review & editing. **S.C. Clarke:** Resources, Writing – review & editing. **S. Srifuengfung:** Resources. **R. Ford:** Resources. **D. Lehmann:** Resources. **E. Egorova:** Resources. **E. Voropaeva:** Resources. **G. Haraldsson:** Resources, Writing – review & editing. **K.G. Kristinsson:** Resources, Writing – review & editing. **L. McGee:** Writing – review & editing. **R.F. Breiman:** Writing – review & editing. **S.D. Bentley:** Writing – review & editing. **C.L. Sheppard:** Resources, Writing – review & editing. **N.K. Fry:** Writing – review & editing. **J. Corander:** Writing – review & editing. **M. Toropainen:** Resources, Writing – review & editing. **A. Steens:** Conceptualization, Writing – review & editing, Project administration.

## The Global Pneumococcal Sequencing Consortium

.

## Declaration of Competing Interest

The authors declare the following financial interests/personal relationships which may be considered as potential competing interests: [R.A.G received a PhD stipend from Pfizer 2009–2011. L.S is a co-investigator in an unrelated study, for which THL has received research funding from GlaxoSmithKline Vaccines. S.C.C: acts as principal investigator on studies conducted on behalf of University Hospital Southampton NHS Foundation Trust/University of Southampton that are sponsored by vaccine manufacturers but receives no personal payments from them. S.C.C. has participated in advisory boards for vaccine manufacturers but receives no personal payments for this work. S.C.C. has received financial assistance from vaccine manufacturers to attend conferences. All grants and honoraria are paid into accounts within the respective NHS Trusts or Universities, or to independent charities. N.K.F, C.L.S The Immunisation and Countermeasures Division, Public Health England - National Infection Service, London, UK provides vaccine manufacturers with post-marketing surveillance reports, which the Marketing Authorization Holders are required to submit to the UK Licensing authority in compliance with their Risk Management Strategy. A cost recovery charge is made for these reports. N.K.F and C.S. conduct contract research funded by vaccine manufacturers (including GlaxoSmithKline and Pfizer) on behalf of Public Health England. No personal remuneration is received. M.T reports grants from GlaxoSmithKline and Pfizer to the Finnish Institute for Health and Welfare for unrelated research projects in which she is a co-investigator. All remaining authors report no conflicts of interest.].
