# Supplementary material for: International links between Streptococcus pneumoniae vaccine serotype 4 sequence type (ST) 801 in Northern European shipyard outbreaks of invasive pneumococcal disease
Source: Vaccine. 2022 Feb 11;40(7):1054–60. doi: 10.1016/j.vaccine.2021.10.046 (PMC8820377; doi:10.1016/j.vaccine.2021.10.046)
Supplement: Supplementary data 2 [file mmc2.docx]

The Global Pneumococcal Sequencing Consortium:

Patrick E Akpaka. Department of Paraclinical Sciences, The University of the West Indies, St. Augustine, Trinidad and Tobago

Krow Ampofo, Division of Pediatric Infectious Diseases, Department of Pediatrics, School of Medicine, University of Utah, 295 Chipeta Way, PO BOX 581289, Salt Lake City, UT, 84108, USA.

Martin Antonio, WHO Collaborating Centre for New Vaccines Surveillance, Medical Research Council Unit The Gambia at The London School of Hygiene & Tropical Medicine, Fajara, The Gambia.

Veeraraghavan Balaji, Christian Medical College, Vellore, India

Bernard W. Beall, Centers for Disease Control and Prevention, Atlanta, USA

Houria Belabbès. Ibn Rochd university-hospital center-Casablanca

Rachel Benisty, The Faculty of Health Sciences, Ben-Gurion University of the Negev, Beer-Sheva, Israel

Stephen D. Bentley, Parasites and microbes, Wellcome Sanger Institute, Hinxton, UK

Godfrey Bigogo, Centre for Global Health Research, Kenya Medical Research Institute, Kisumu, Kenya

Robert F. Breiman, Rollins School Public Health, Emory University, USA; Emory Global Health Institute, Atlanta, USA.

Abdullah W Brooks. International Centre for Diarrheal Diseases Research, Dhaka, Bangladesh

Philip E. Carter. Institute of Environmental Science and Research Limited, Kenepuru Science Centre, Porirua, Zealand

Stuart C Clarke. Faculty of Medicine and Institute of Life Sciences, University of Southampton, UK

Jennifer E. Cornick, Malawi-Liverpool-Wellcome-Trust, Malawi

Alejandra Corso. Instituto Nacional de Enfermedades Infecciosas, Argentina

Maria Cristina de Cunto Brandileone, Center of Bacteriology, Adolfo Lutz Institute, São Paulo, Brazil

Samanta Cristine Grassi Almeida, Center of Bacteriology, Adolfo Lutz Institute, São Paulo, Brazil

Nicholas J. Croucher, Faculty of Medicine, School of Public Health, Imperial College London, UK.

Ron Dagan, The Faculty of Health Sciences, Ben-Gurion University of the Negev, Beer-Sheva, Israel

Alexander Davydov. Belarusian State Medical University, Minsk, Belarus; The Republican Research and Practical Center for Epidemiology and Microbiology, Minsk, Belarus

Idrissa Diawara. Faculty of Sciences and Techniques of Health, Mohammed VI University of Health Sciences (UM6SS).

Sanjay Doiphode. Hamad Medical Corporation, Doha, Qatar

Mignon du Plessis, Centre for Respiratory Diseases and Meningitis, National Institute for Communicable Diseases, Johannesburg, South Africa

Ekaterina Egorova. Gabrichevsky Epidemiology and Microbiology Research Institute, Moscow, Russia

Naima Elmdaghri. Laboratoire of Microbiology, Faculty of Medicine and Pharmacy & Ibn Rochd University Hospital Center, Casablanca, Morocco.

Özgen Köseoglu Eser. Hacettepe University Faculty of Medicine, Department of Medical Microbiology, 06100, Ankara, Turkey

Dean B. Everett, Queens Research Institute, University of Edinburgh, UK

Diego Faccone. Instituto Nacional de Enfermedades Infecciosas, Argentina

Rebecca Ford. Papua New Guinea Institute of Medical Research, PO Box 60, Goroka, 441, Eastern Highlands Province, Papua New Guinea

Paula Gagetti. Instituto Nacional de Enfermedades Infecciosas, Argentina

Noga Givon-Lavi. The Faculty of Health Sciences, Ben-Gurion University of the Negev, Beer-Sheva, Israel,

Rebecca A. Gladstone, Parasites and microbes, Wellcome Sanger Institute, Hinxton, UK;Department of Biostatistics, University of Oslo, Norway

Md Hasanuzzaman, Child Health Research Foundation, Department of Microbiology, Dhaka Shishu Hospital, Dhaka 1207, Bangladesh

Paulina A. Hawkins, Rollins School Public Health, Emory University, USA

Waleria Hryniewicz, ^10^National Medicines Institute, Division of Clinical Microbiology and Infection Prevention, Warsaw, Poland

Kristina G. Hulten, Department of Pediatrics, Baylor College of Medicine, Houston TX

Margaret Ip. Dept of Microbiology, Chinese Univ of Hong Kong

Aurelie Kapusta, Department of Human Genetics, University of Utah, 15 North 2030 East, Salt Lake City, UT 84112

Rama Kandasamy. Oxford Vaccine Group, Department of Paediatrics, University of Oxford, and the NIHR Oxford Biomedical Research Centre, Oxford, UK

Tamara Kastrin. Department of Medical Microbiology, Institute of Public Health of the Republic of Slovenia, Grabloviceva 44, 1000 Ljubljana, Slovenia

Jeremy Keenan, Francis I. Proctor Foundation, University of California, San Francisco, San Francisco, California, United States of America

Keith P. Klugman, Rollins School Public Health, Emory University, USA

Brenda Kwambana-Adams, NIHR Global Health Research Unit on Mucosal Pathogens, Division of Infection and Immunity, University College London, London, UK; WHO Collaborating Centre for New Vaccines Surveillance, Medical Research Council Unit The Gambia at The London School of Hygiene & Tropical Medicine, Fajara, The Gambia.

Pierra Y. Law, Department of Microbiology and Carol Yu Centre for Infection, The University of Hong Kong, Queen Mary Hospital, Hong Kong, China

John A Lees^,^ Faculty of Medicine, School of Public Health, Imperial College London, UK.

Deborah Lehmann. Telethon Kids Institute, the University of Western Australia, Perth, WA

Pak Leung Ho, Department of Microbiology and Carol Yu Centre for Infection, The University of Hong Kong, Queen Mary Hospital, Hong Kong, China

Yuan Li, Centers for Disease Control and Prevention, Atlanta, USA

Stephanie W. Lo, Parasites and microbes, Wellcome Sanger Institute, Hinxton, UK

Theresa J. Ochoa, Instituto de Medicina Tropical, Universidad Peruana Cayetano Heredia, Lima, Peru

Shabir A. Madhi, Medical Research Council: Respiratory and Meningeal Pathogens Research Unit, University of the Witwatersrand, South Africa; Dept. of Science and Technology/National Research Foundation: Vaccine Preventable Diseases, University of the Witwatersrand, South Africa

Lesley McGee, Centers for Disease Control and Prevention, Atlanta, USA

Benjamin J Metcalf, Centers for Disease Control and Prevention, Atlanta, USA

Jennifer Moïsi. Agence de Médecine Préventive, Paris, France

Helio Mucavele Fundação Manhiça, Centro de Investigação em Saúde da Manhiça (CISM), Maputo, Moçambique

Kedibone M. Ndlangisa, Centre for Respiratory Diseases and Meningitis, National Institute for Communicable Diseases, Johannesburg, South Africa

Michele Nurse-Lucas. Department of Paraclinical Sciences, The University of the West Indies, St. Augustine, Trinidad and Tobago

Susan A. Nzenze, Medical Research Council: Respiratory and Meningeal Pathogens Research Unit, University of the Witwatersrand, South Africa; Dept. of Science and Technology/National Research Foundation: Vaccine Preventable Diseases, University of the Witwatersrand, South Africa

Stephen K Obaro. University of Nebraska Medical Center, Omaha, USA

Metka Paragi. National Laboratory of Health, Environment and Food, Centre for Medical Microbiology, Department for Public Health Microbiology, Grablovičeva 44, 1000, Ljubljana, Slovenia

Andrew J Pollard, Oxford Vaccine Group, Department of Paediatrics, University of Oxford, and the NIHR Oxford Biomedical Research Centre, Oxford, UK

KL. Ravikumar, Central Research Laboratory, Department of Microbiology, Kempegowda Institute of Medical Sciences Hospital & Research Center, Bangalore, India

Ewa Sadowy. Department of Molecular Microbiology, National Medicines Institute, 00-725 Warsaw, Poland

Samir K. Saha, Child Health Research Foundation, Dhaka, Bangladesh

Eric Sampane-Donkor, Department of Medical Microbiology, School of Biomedical and Allied Health Sciences University of Ghana, Accra, Ghana

Shamala Devi Sekaran. Faculty of Medical & Health Sciences, UCSI University, Malaysia

Sadia Shakoor. Department of Pathology and Laboratory Medicine and Department of Paediatrics and Child Health, The Aga Khan University, Karachi 74800, Pakistan.

Shrijana Shrestha, Patan Academy of Health Sciences, Kathmandu, Nepal

Betuel Sigauque, Fundação Manhiça / Centro de Investigação em Saúde da Manhiça (CISM); Maputo, Mozambique, Instituto Nacional de Saúde, Ministério de Saúde, Maputo, Mozambique

Anna Skoczynska. National Reference Centre for Bacterial Meningitis, Department of Epidemiology and Clinical Microbiology, National Medicines Institute, Warsaw, Poland

Kwan Soo ko. Department of Molecular Cell Biology, Samsung Biomedical Research Institute, Sungkyunkwan University School of Medicine, Suwon, South Korea

Somporn Srifuengfung. Department of Microbiology, Faculty of Medicine Siriraj Hopital, Mahidol University, Bangkok, Thailand.

Peggy-Estelle Tientcheu. Vaccines and Immunity Theme, MRC Unit, The Gambia

Leonid Titov. The Republican Research and Practical Center for Epidemiology and Microbiology, Minsk, Belarus

Paul Turner. Centre for Tropical Medicine and Global Health, Nuffield Department of Medicine, University of Oxford, Oxford, UK.

Yulia Urban. Gabrichevsky Epidemiology and Microbiology Research Institute, Moscow, Russia

Jennifer Verani, Respiratory Diseases Branch, National Center for Immunization and Respiratory Diseases, Centers for Disease Control and Prevention, Atlanta, United States of America

Andries J. van Tonder, Department of Veterinary Medicine, University of Cambridge, Cambridge, UK

Anne von Gottberg, Centre for Respiratory Diseases and Meningitis, National Institute for Communicable Diseases, Johannesburg, South Africa

Elena Voropaeva. Gabrichevsky Epidemiology and Microbiology Research Institute, Moscow, Russia

Nicole Wolter. Centre for Respiratory Diseases and Meningitis, National Institute for Communicable Diseases, Johannesburg, South Africa
